# Task-Related Modulations of BOLD Low-Frequency Fluctuations within the Default Mode Network

**DOI:** 10.3389/fphy.2017.00031

**Published:** 2017-07-25

**Authors:** Silvia Tommasin, Daniele Mascali, Tommaso Gili, Ibrahim Eid Assan, Marta Moraschi, Michela Fratini, Richard G. Wise, Emiliano Macaluso, Silvia Mangia, Federico Giove

**Affiliations:** 1 MARBILab, Centro Fermi—Museo Storico Della fisica e Centro Studi e Ricerche Enrico Fermi, Rome, Italy; 2 Fondazione Santa Lucia IRCCS, Rome, Italy; 3 Dipartimento di Fisica, Sapienza Università di Rome, Rome, Italy; 4 Istituto di Nanotecnologia, Consiglio Nazionale delle Ricerche, Rome, Italy; 5 Cardiff University Brain Research Imaging Centre, School of Psychology, Cardiff University, Cardiff, United Kingdom; 6 ImpAct Team, Lyon Neuroscience Research Center, Lyon, France; 7 Center for Magnetic Resonance Research, University of Minnesota Twin Cities, Minneapolis, MN, United States

**Keywords:** low frequency BOLD fluctuations, functional connectivity, DMN, working memory, fALFF

## Abstract

Spontaneous low-frequency Blood-Oxygenation Level-Dependent (BOLD) signals acquired during resting state are characterized by spatial patterns of synchronous fluctuations, ultimately leading to the identification of robust brain networks. The resting-state brain networks, including the Default Mode Network (DMN), are demonstrated to persist during sustained task execution, but the exact features of task-related changes of network properties are still not well characterized. In this work we sought to examine in a group of 20 healthy volunteers (age 33 ± 6 years, 8 F/12 M) the relationship between changes of spectral and spatiotemporal features of one prominent resting-state network, namely the DMN, during the continuous execution of a working memory n-back task. We found that task execution impacted on both functional connectivity and amplitude of BOLD fluctuations within large parts of the DMN, but these changes correlated between each other only in a small area of the posterior cingulate. We conclude that combined analysis of multiple parameters related to connectivity, and their changes during the transition from resting state to continuous task execution, can contribute to a better understanding of how brain networks rearrange themselves in response to a task.

## INTRODUCTION

Spontaneous low-frequency fluctuations (LFFs) of the BOLD signal are thought to be a manifestation of the ongoing activity of the brain [1]. Such BOLD LFFs are spatially synchronized in the brain, identifying robust and reproducible connectivity patterns also known as resting-state networks [2]. Although, BOLD LFFs have been subject of intense research, the relationship between network connectivity and spectral features of the signal is still not completely characterized. Even less clear is how this relationship is influenced by the cognitive activity of the brain.

Of particular interest is the characterization of the spontaneous activity within the DMN, that is considered an intrinsic property of the human brain, relevant for brain physiology and pathology [3]. The specific interest for DMN is justified because features of slow BOLD fluctuations within the DMN have been linked to multiple brain processes (recently reviewed by [4]). Indeed, cognition has been shown to depend on the activity of the DMN itself [5], as well as on the connectivity between DMN and other networks, such as the “task–positive” network [6]. Far from being a static property of the brain, connectivity within DMN and between DMN and other networks is influenced by behavior [7].

Task-related modulations of the DMN are not restricted to effects on connectivity. Indeed, the amplitude of BOLD LFFs within the DMN depends on the cognitive activity, decreasing during a working memory task [8] or after a continuous and prolonged attention task [9]. Level of activity in the DMN has been found to be modulated by working memory load both during and after task performance [10, 11]. The amplitude of LFFs has been proposed as a measure to assess the degree of intrinsic brain activity, offering insights into the physiological determinants of functional connectivity [12, 13]. Various metrics have been proposed to assess BOLD fluctuation amplitude, including ALFF (amplitude of LFF, [13, 14]) and its normalized version fALFF (fractional ALFF), less sensitive to noise [15]. ALFF has been found to be altered in several pathologies [14, 16, 17], and to be also influenced by the behavioral state or by the task [18, 19]. Moreover, the amplitude of fluctuations at rest has been shown to correlate with brain activation and deactivation in DMN during the execution of an N-back working memory task, and this correlation was found to be load dependent [20]. The load dependency of the relationship between rest and task fluctuations suggests that the involved regions are characterized by both a great capacity for enhancing flow and metabolism under stimulation, and a large fluctuation amplitude at rest.

The spectral amplitude of BOLD LFFs is often integrated over a relatively broad range (typically between 0.01 and 0.1Hz), however it has been suggested that a finer spectral subdivision can allow a better grasp of the underlying physiological phenomena [21–23]. In particular, ALFF within the bands labeled slow-5 (0.01–0.027Hz) and slow-4 (0.027–0.073Hz) brings most of the neuronal-related information (compared to higher frequency ranges) and is characterized by distinct spatial patterns [23, 24].

BOLD response signals show a spatially segregated coupling with electrophysiological signals (e.g., [25–27]) and metabolism [28, 29], yet the connectivity patterns are only partially determined by anatomic constraints [30]. Brain spontaneous activity, embodied in functional connectivity, has been shown to account to up to 70% of the energy consumed by the brain [31], therefore, the local relationship between fluctuation amplitude and functional connectivity has the potential to clarify some basic features of functional networks. Accordingly, correlation between oscillation amplitude and functional connectivity has been found in healthy subjects at rest [32], and was reported to be decreased in Alzheimer’s disease patients [33]. Moreover, the temporal variance of dynamic local functional connectivity has been associated with the temporal variance of ALFF [34], and regional synchrony of BOLD fluctuations has been proposed as a determinant for neurovascular coupling variability [35].

In the present study, we sought to elucidate whether the relationship between amplitude and connectivity strength of BOLD fluctuations within the DMN can be modulated by behavior. For this purpose, we quantified spectral and spatiotemporal features of BOLD LFFs within the DMN during resting state and during the sustained execution of a graded working memory task.

## MATERIALS AND METHODS

### Subjects

Twenty healthy Italian–speaking subjects (age 33 ± 6 years, 8 female) participated in the present study. The study was carried out in accordance with a protocol approved by the Ethics Committee of Santa Lucia Foundation in Rome. Recruited subjects gave written informed consent in accordance with the Declaration of Helsinki and European Union regulations.

### Image Acquisition

Data were collected on a 3 T MRI system (Magnetom Allegra, Siemens Healthineers, Erlangen, Germany). Functional images were acquired via 2D gradient-echo planar sequence (TE = 30 ms, TR = 2,100 ms, FA = 70°, voxel size 3 × 3 × 2.5 mm^3^) lasting 24min and 38 s for a total of 704 volumes (4 dummy scans included). Sagittal, T_1_-weighted structural data were acquired for tissue segmentation purpose (MPRAGE, TE = 4.38ms, TR = 2,000 ms, FA = 8°, voxel size 1.33 × 1.33 × 1 mm^3^).

### Stimulation Paradigm

During the functional runs, subjects were presented with a stimulation paradigm consisting of alternated epochs of open-eyes resting state and sustained auditory working memory task (4min and 54 s each, starting with resting-state epoch). The auditory working memory task involved continuous n-back trials administered in epochs either at “high” load (2-back) or “low” load (1-back). Each trial was composed of a 500-ms window, in which subjects were aurally presented with a pseudorandom vowel (A, E, or O), and a subsequent 1,600-ms window, in which subjects were asked to press a button every time the current vowel was the same as the one presented one stimulus prior (1-back) or two stimuli prior (2-back).

Two functional runs were acquired for each subject during the same experimental session, with epoch ordering: rest/1-back/rest/2-back/rest and rest/2-back/rest/1-back/rest. Run order was counterbalanced across subjects. The stimulation paradigm started after the second dummy scan (i.e., 2 scans before the first analyzed image) to roughly account for hemodynamic delay.

### Image Processing

Functional and structural MRI data were preprocessed using functional connectivity toolbox version 15.b [36] and analyzed with dedicated in-house routines based on MATLAB R2013a (The Mathworks, Natick, MA, USA) and AFNI [37]. T_1_ weighted images were segmented in white matter (WM) and Cerebrospinal Fluid (CSF) probability maps to be later used for denoising. Functional data underwent standard preprocessing, including removal of the first four volumes of each fMRI run, realignment, slice-timing correction, normalization to MNI space (using as source volume the mean EPI image) and spatial smoothing with an isotropic Gaussian kernel (8 × 8 × 8 mm^3^ FWHM). Several spurious sources of variance were removed from smoothed functional data via regression analysis, including the estimated realignment parameters and their first derivative, signals from WM and CSF following the aCompcor approach [38] and outlier volumes detected using the Artifact Detection Tools (ART: www.nitrc.org/projects/artifactdetect/). Finally, a band-bass temporal filter in the range 0.008–0.09 Hz was applied to the residual time-series. An unfiltered time-series was also retained for fALFF computation.

Each functional run was then split in its five epochs, which were later used to extract epoch-related functional parameters (see below). A DMN mask was derived by independent component analysis (FSL MELODIC toolbox, [39]) of the first resting-state epoch and was used to constrain the subsequent analyses. The first resting-state epoch was then discarded, leaving 2 resting-state and 2 task epochs for each of the two functional runs.

A second dataset was obtained with the same procedures, but with isotropic Gaussian kernel at 6 × 6 × 6mm^3^ FWHM for testing purposes. All the following results are referred to the 8^3^ mm^3^ smoothing dataset, unless otherwise stated.

### Computation of Parameters

Each of the following parameters was computed separately in each functional epoch, thus they represent specific features of BOLD LFFs at specific steady-state conditions.

As a measure of average network strength, within-network functional connectivity (FC) was evaluated as the Pearson’s correlation coefficient between each voxel’s time course and the average time course of the whole DMN. Correlation maps were z-Fisher transformed to improve normality.

To quantify the amplitude of LFFs, fALFF was calculated for each voxel time series as the summation of the spectral amplitude in the selected low–frequency range (between 0.008 and 0.09Hz) divided by the summation in the full frequency range [15]. The computation was performed via the AFNI program 3dRSFC [37]. Average contribution of different frequency bands was also evaluated by estimating the Power Spectral Density (PSD) of the average BOLD time course within the DMN. The PSD was estimated via the squared magnitude of the Fast Fourier Transform of the signal, and averaged separately in the slow-5 (0.01–0.027Hz) and slow-4 (0.027–0.073Hz) bands. PSD was also normalized by its integrated power.

### Analysis of Changes Associated with Sustained Working Memory Task

Stationary changes of each investigated parameter (fALFF and FC) between task and the resting epoch immediately following it were evaluated voxel by voxel (irrespectively of the task level) and tested for significance by paired *t*-test across subjects, after averaging separately the parameters of interest across the two functional runs of each subject.

Changes of the PSD profile were assessed by linearly fitting the normalized PSD of each epoch, and testing the resulting slopes via repeated measures ANOVA and *post-hoc* Bonferroni corrected paired *t-*test. Task-related changes of PSD integrated magnitude were assessed by 2-way repeated measures ANOVA and *post-hoc t*-tests (Bonferroni corrected), defining as factors the stimulation condition (rest, 1-back, 2-back) and the frequency band (slow-5 and slow-4).

Finally, to test whether changes of functional connectivity are related to changes of amplitude of fluctuations we computed voxelwise Pearson correlation of the relevant quantities across subjects.

Statistical threshold for voxelwise comparisons was set to *p* < 0.05 corrected for multiple comparisons at cluster level by Monte Carlo simulation (AFNI, 3dClustSim). The corrected threshold corresponded to a single-voxel *p* < 0.001 and a minimum cluster size depending on the estimated smoothness of fit residuals and on the number of voxels within the DMN mask. Smoothness of fit residuals was estimated using a mixed model for autocorrelation function of noise [40, 41].

## RESULTS

**FIGURE 1:**
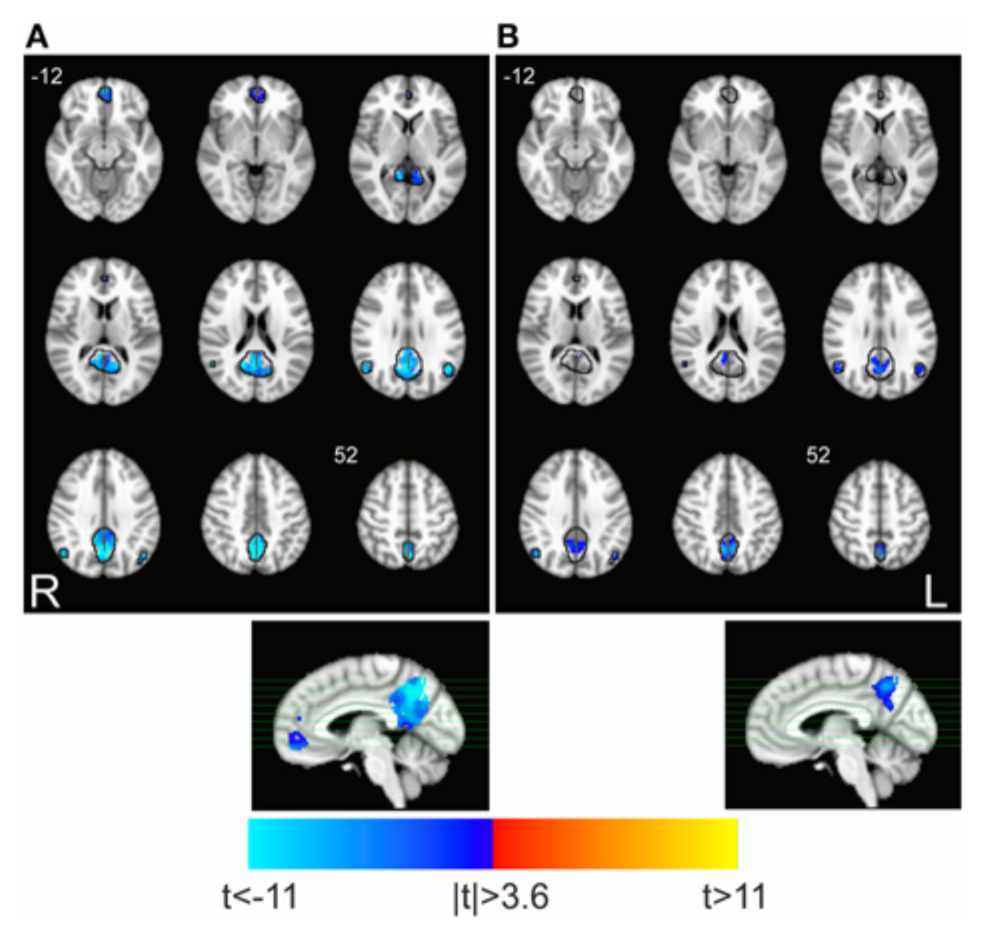
Task-related changes in spectral and spatio-temporal parameters within DMN. Maps show the significant task-associated changes of **(A)** fALFF and **(B)** FC for the test TASK > REST (paired *t*-test, *p* < 0.05 corrected. 3dClustSim parameters: single-voxel *p* < 0.001; cluster size threshold, 10 and 18 voxels, for fALFF and FC, respectively). Black lines identify the boundaries of DMN as identified by ICA, where analysis was restricted. Task-related decrements of fALFF and FC are apparent in the posterior DMN, and in the anterior cingulate only for fALFF.

fALFF and FC were significantly reduced within posterior areas of DMN during task execution, including the precuneus (bilaterally) and the posterior division of the cingulate (Figures 1A, B). The anterior portion of DMN showed significant task-related fALFF changes in the medial prefrontal cortex, but did not show any significant change of FC. Very similar results were obtained on the 6 × 6 × 6 mm^3^ smoothing dataset (not shown).

**FIGURE 2:**
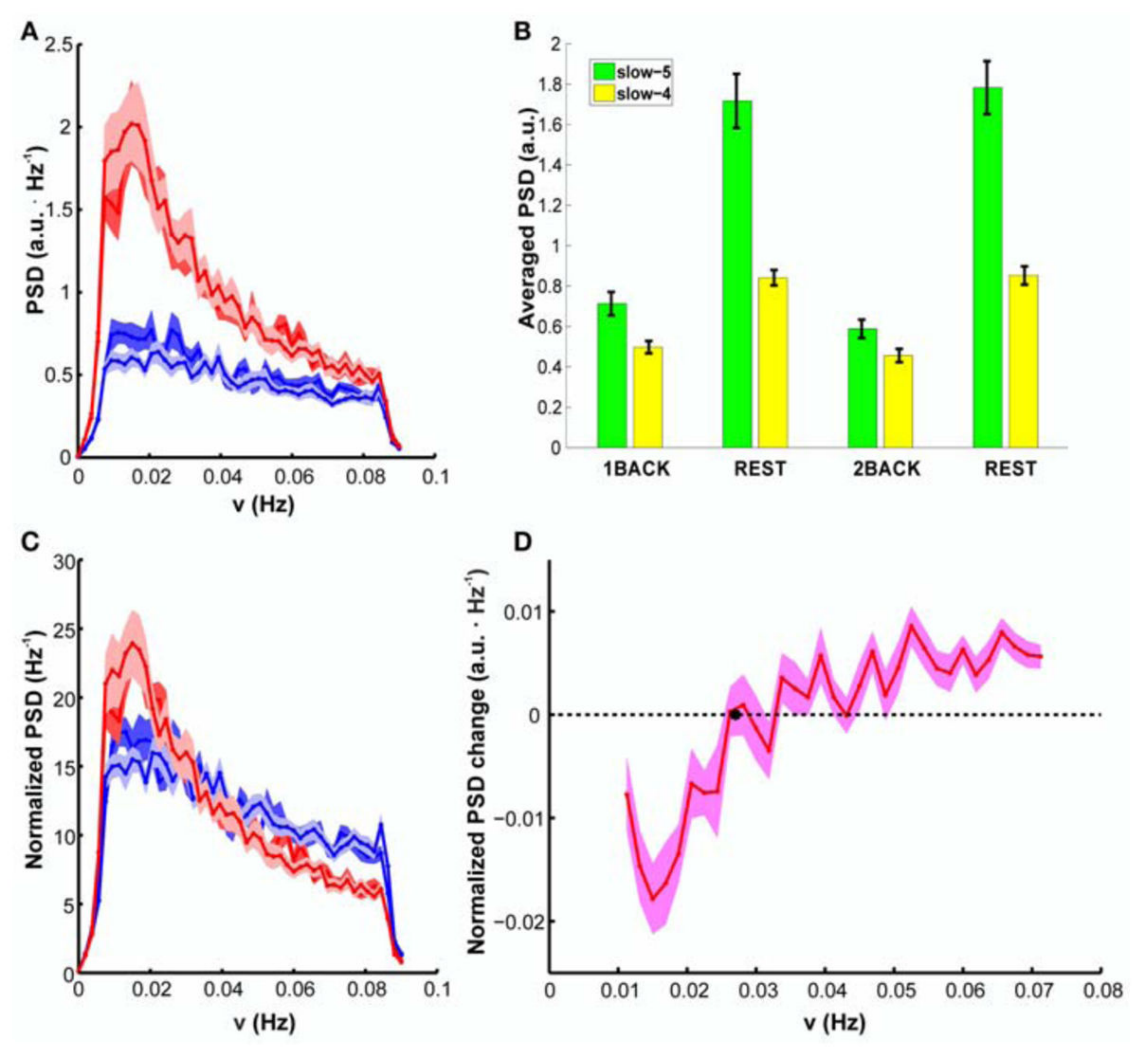
PSD group results: **(A)** PSD of BOLD fluctuations in the DMN, averaged in each epoch. Resting-state epochs are represented in red, task epochs in blue (dark blue is 1 back, light blue is 2 back). Task-related changes in spectral power are visible. **(B)** PSD averaged in the slow-4 (green) and slow-5 (yellow) range. Slow-5 presents higher integrated power in all epochs. Power was always higher at rest than during task, but in a frequency specific manner (significant interaction stimulation condition x frequency band). The two task levels were indistinguishable. See Results Section for *p*-values. **(C)** Normalized PSD. In normalized PSD a steeper dependence on frequency during resting state than during task performance is apparent. **(D)** Difference between normalized PSD during task execution and resting state as a function of frequency. All values are mean ± SEM across subjects. SEM is computed after within-subject averaging of corresponding data from the two runs.

Spectral analysis revealed that the task-related fALFF decrement occurs with a reduction of fluctuations power at each investigated frequency (Figure 2A). Comparison of error bands suggests that PSD of BOLD fluctuations is very reproducible between sessions at rest (compare light red and dark red lines in Figure 2A). Any possible effect of task level is confined to the lower part of frequency spectrum (below 0.02Hz, light and dark blue lines in Figure 2A, see also Supplementary Figure 1 for the confidence band of the PSD difference between task levels). Values averaged within slow-5 and slow-4 bands confirmed the result, showing a consistent task-related decrease of PSD, but no average effect of task level [Figure 2B, 2-way ANOVA, *F*_(3, 57)_ > 85, *p* < 0.001 for the factor “stimulation condition”; *post-hoc* Bonferroni paired *t-*test *t* > 10, *p* < 0.001, *n* = 20 for task vs. rest; *t* < 1.4, *p* > 0.9, *n* = 20 for differences between 1-back and 2-back].

The task-related decrement of fluctuation power was not uniform across the whole spectrum. Indeed, the reduction of PSD magnitude during task was less marked at the higher frequencies, both in absolute terms and in terms of fractional change compared to rest (compare changes in slow-4 and slow-5 bands, Figure 2B). This feature was demonstrated by a significant interaction between factors (stimulation condition and frequency band) in 2-way ANOVA [*F*_(3, 57)_ > 32, *p* < 0.001]. Analysis of simple main effects confirmed that, in both bands, power change in task vs. rest comparison was always significant, and task (respectively rest) epochs were indistinguishable among them (*p* < 0.001 for all significant comparisons, *p* > 0.8 for all not significant comparisons). Accordingly, the normalized PSD highlighted an higher power contribution from the lower frequencies, but it was characterized by a steeper decrease of power with frequency during rest than during task; in other words, the normalized PSD became significantly flatter during task (Figure 2C). The change of normalized PSD shape between treatments was also confirmed by repeated measures ANOVA on the average slope [*F*_(3, 57)_ > 46, *p* < 0.001; Table 1]; relevant *post-hoc* tests indicated that the LFFs normalized spectrum was remarkably reproducible between rest epochs (*t* < 1.3, *p* > 0.9, *n* = 20) and between task epochs, irrespectively of the task level (*t* < 1.8, *p* > 0.4, *n* = 20). The slope was however reduced by task (*t* > 6.6, *p* < 0.001, *n* = 20 for all comparisons).

In agreement with this finding, the difference between the pooled task and rest conditions showed an overall increase with frequency (Figure 2D); interestingly, the difference crosses zero (thus the normalized PSD at rest and task are equal) at a frequency roughly compatible with the boundary between slow-4 and slow-5 bands.

**FIGURE 3:**
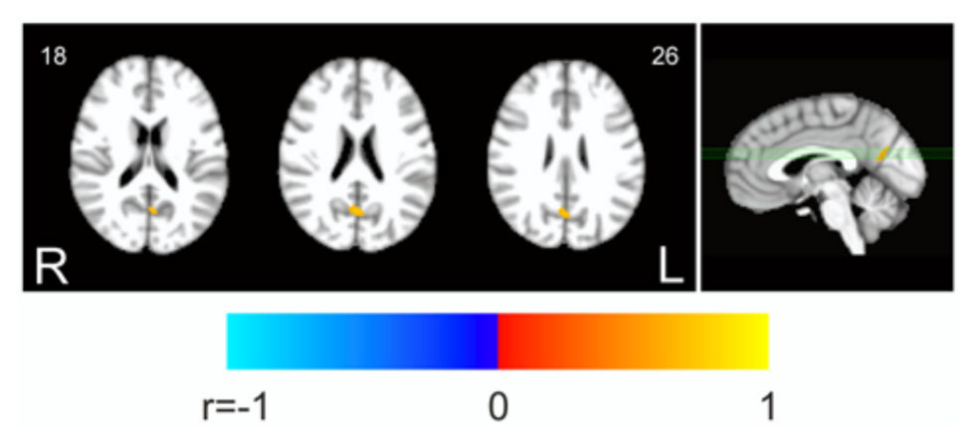
Correlation between fALFF and FC changes within the DMN. The map shows the voxelwise thresholded results of correlation analysis between task-related changes of fALFF and FC. Decrease of fALFF was correlated to decrease of FC in a small single cluster within the posterior DMN areas (*p* < 0.05 corrected. 3dClustSim parameters: single-voxel *p* < 0.001; cluster size threshold, 16 voxels).

Finally, the voxel-wise analysis within the DMN revealed that the decrement of FC (Figure 1A) was correlated to the decrease of fALFF (Figure 1B) in a small area in the posterior cingulate (Figure 3, unthresholded correlation map is reported in Supplementary Figure 2). Equivalent results were obtained on the 6 × 6 × 6 mm^3^ smoothing dataset (not shown).

## DISCUSSION

In this work we sought to test whether changes in connectivity during a working memory task are mirrored by changes in fluctuation amplitude within the DMN, and if task-related changes of the two parameters are correlated. We found a remarkably homogeneous reduction of both fALFF and FC during sustained working memory task in the posterior areas of DMN, while changes in the anterior cingulate were less marked (Figures 1A, B).

Task-related reductions in fluctuation amplitude, as indexed by fALFF metric, were also evident in the power spectrum within the DMN mask. Task epochs showed a pronounced reduction in power at each investigated frequency, in a consistent and reproducible manner across different task loads (Figure 2A). However, the decrease in fluctuation power within the DMN was not uniform across the explored frequency range. Indeed, while both rest and task states showed higher fluctuation power in the slow-5 band compared to the slow-4 band, consistently with the known 1/f power distribution of BOLD LFFs [42, 43], the trend was less marked during tasks (Figure 2B). Accordingly, normalized spectral power showed state-dependent trends for slow-5 and slow-4 bands, suggesting that the switch to task state has a major impact on the lower frequency component, or (possibly) that the lower frequency component is more relevant to the switch of brain function.

The reduced power of LFFs during the task execution is in agreement with previous studies [8, 43, 44]. Indeed, power distributions of both task and rest states have shown to follow a power law, with significant reduced exponent during task states [43], compatible with the task-related decrease of PSD we reported. Moreover, reduced BOLD fluctuations were observed within the DMN during a working memory task, which, similarly to our results, were more notable in posterior regions [8]. At odds with our results, Zhang et al. have reported mainly decrements of fALFF during a stop signal task within the DMN [45]. While several factors might contribute to explain the discrepancy with our results (e.g., different task condition and the use of task-residual instead of a continuous and prolonged acquisition of task state), more likely it might be explained by the transformation of fALFF in z-score which removes the mean difference across states.

The reduction in fluctuation amplitude mirrors the tendency of the DMN to reduce its spontaneous activity during cognitive engagement. This result shows that task execution affects brain regions within the DMN inducing both a time-locked functional deactivation, as shown in countless task-based experiments, and a modulation of spontaneous fluctuations toward low level of activity. In addition, several studies have demonstrated that DMN deactivation increases with cognitive load [10, 11] indicating that cognitive resources are reallocated according to task demand and that endogenous processes need to be inhibited at different levels for successful execution. This could be interpreted as a redistribution of cognitive ability but also as a neuronal correlate of mental fatigue [11], self-referential processes [46], or mind-wandering [47]. In our case, aggregate values of LFFs power spectral density within slow-4 and slow-5 bands (Figure 2B) did not reveal any effect of load, while spectral decomposition suggested that any effect of load is confined to the lowest spectral range, below 0.02Hz (about half of the slow-5 band, Figure 2A and Supplementary Figure 1). These results suggest that amplitude of fluctuations and DMN deactivation react differently to an increase of cognitive activity. Incidentally, the inhomogeneous response of PSD to task within the slow-5 band suggests that a further subdivision of slow-5 into 2 or more bands can help to identify subtle frequency-specific effects on the amplitude of BOLD LFFs.

Functional connectivity and ALFF during resting state have shown region specific couplings in elderly populations [32, 33] which were suggested to be of physiological relevance being disrupted in degenerative dementia [33]. While these studies have shown inter-subject associations between connectivity and amplitude at rest, others have also found within-subject synchronized fluctuations of the two parameters [34]. We found that the task-related decreases of fALFF and FC are highly correlated within a small area of the DMN(Figure 3), supporting a physiological relation between the two parameters, even within the DMN, which is not directly involved in the execution of the task. This result suggests that a change of functional connectivity reflects a change of amplitude of fluctuations at least in spatially segregated regions within posterior DMN areas. Taking into account a connection between amplitude of fluctuations and underlying electrophysiological activity [25, 27], this result overall suggests the neural origin of changes of functional connectivity within the DMN.

The correlation between variation of FC and fALFF within the DMN was not observed uniformly across the whole network. We hypothesize that this finding is related to an intrinsic heterogeneity of the coupling between FC and fALFF changes. Indeed, unthresholded data (Supplementary Figure 2) showed a smooth change of correlation with some local maxima (of which only one exceeds statistical threshold). This feature suggests spatially segregated coupling between FC and fALFF changes. However, the fact that the effect is confined in the areas of most significant FC and fALFF changes (compare Figures 1A, B with Figure 3) may also indicate a possible lack of sensitivity of our experimental design. The lack of generalized correlation between FC and fALFF changes also implies that fALFF changes are not the only determinants of FC changes, a notion that confirms the utility of exploiting both parameters in functional connectivity studies. An intriguing hypothesis, that deserves further analysis, is that the changes of connectivity and of fluctuation amplitude do not share the same spectral sensitivity profile. Indeed, in a previous study we reported band-specific spatial patterns of correlation between FC and ALFF at rest, even if the effect was mainly present outside the DMN [33]. However, in the present study, we found that the amplitude of fluctuation is substantially more affected by task execution in the lower range of frequencies (the slow-5 band), suggesting that, differently for the coupling during resting states, a frequency specific relationship may exist. This hypothesis is also in line with previous resting-state studies that have repeatedly shown frequency-specific alterations in fluctuation amplitude [16, 48, 49] and connectivity [48] during pathological states.

In conclusion, in this work we reported that amplitude and connectivity of BOLD low-frequency fluctuations within the DMN are affected by the sustained performance of a cognitive task. With the exception of the very low frequency component, different cognitive loads were associated with similar modulations in fluctuation amplitude. Task-related modulations of amplitude of fluctuations and connectivity strength are not independent within the DMN. The results we obtained suggest that the correlation between amplitude of BOLD fluctuations and connectivity strength can be exploited to gather insights into the physiology of brain function.

## Supplementary Material

Suppl. Figure 1 Supplementary Figure 1 PSD difference between 1 back and 2 back. The figure reports the 95% confidence band for the difference of LFFs power spectral density between task levels 1-back and 2-back. The confidence band does not overlap zero only below 0.02 Hz.

Suppl. Figure 2 Supplementary Figure 2 Correlation between fALFF and FC changes within the DMN, unthresholded. Unthresholded map of the correlation between fALFF and FC changes within the DMN (unthresholded version of Figure 3). Correlation was generally around 0, except some clusters of positive correlation. Only one of them reached statistical significance (highlighted by black outline, corresponding to Figure 3).

## Figures and Tables

**TABLE 1:** Slope of normalized PSD.

	1-back	Rest after 1-back	2-back	Rest after 2 back
Slope	−0.284	−0.468	−0.235	−0.503
SEM	0.027	0.026	0.021	0.033

Values of the slope of normalized PSD averaged across subjects, separately calculated during task epochs and during rest epochs immediately following each task epoch. The shape is significantly flatter during task than rest [repeated measures ANOVA, F_(3, 57)_ > 46, p < 0.001 between treatments]. Slopes are consistent across resting epochs, as well as across task epochs (Bonferroni paired t-test, t < 1.3, p > 0.9, n = 20 and t < 1.8, p > 0.4, respectively; n = 20). Slopes are reduced between task and the immediately following rest epoch (t > 6.6 p < 0.001, n = 20 and t > 9.6, p < 0.001, n = 20 for 1-back and 2-back, respectively). Slopes are also reduced between task and the chronologically unmatched rest (t > 8.4, p < 0.001, n = 20 for 2-back vs. rest after 1 back, and t > 7.8, p < 0.001, n = 20 for 1-back vs. rest after 2 back). SEM, Standard Error of the Mean.
