# Supplementary figures and images for: Task-Related Modulations of BOLD Low-Frequency Fluctuations within the Default Mode Network
Source: Front Phys. Author manuscript; Available in PMC 2017 Aug 23. (PMC5568127; doi:10.3389/fphy.2017.00031)

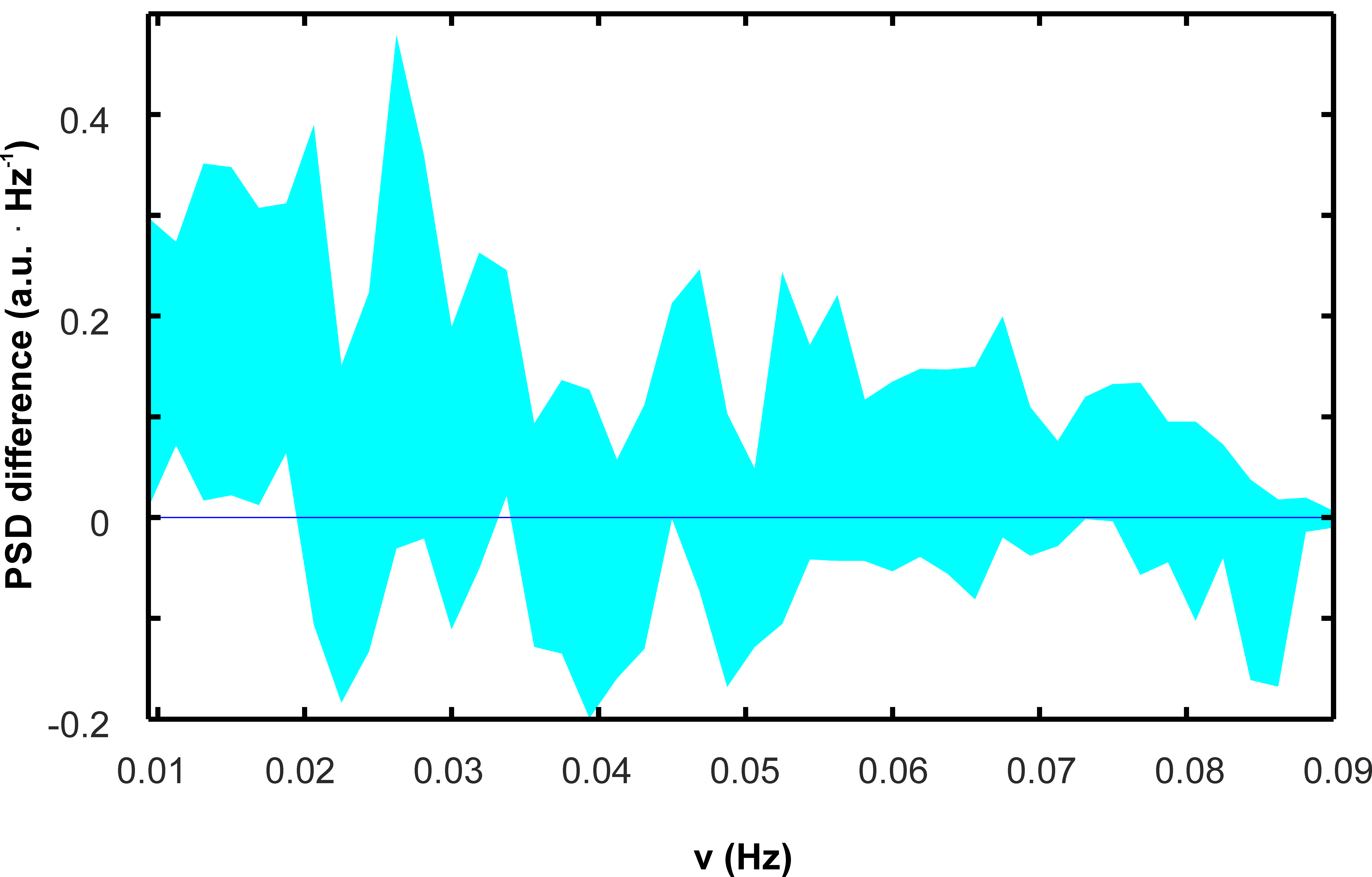

Supplement: Suppl. Figure 1 — PSD difference between 1 back and 2 back. The figure reports the 95% confidence band for the difference of LFFs power spectral density between task levels 1-back and 2-back. The confidence band does not overlap zero only below 0.02 Hz. [file NIHMS895351-supplement-Suppl__Figure_1.png]

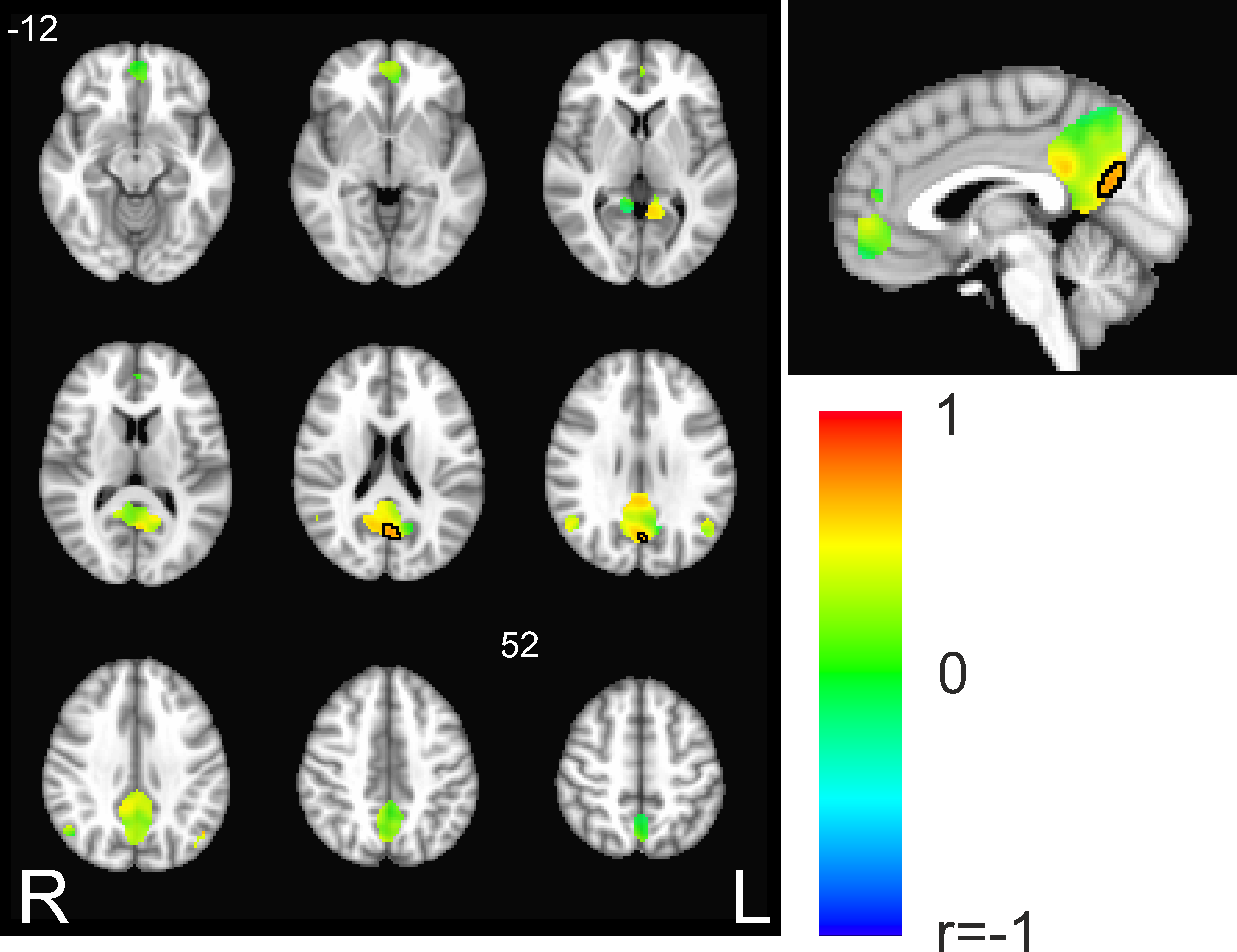

Supplement: Suppl. Figure 2 — Correlation between fALFF and FC changes within the DMN, unthresholded. Unthresholded map of the correlation between fALFF and FC changes within the DMN (unthresholded version of Figure 3). Correlation was generally around 0, except some clusters of positive correlation. Only one of them reached statistical significance (highlighted by black outline, corresponding to Figure 3). [file NIHMS895351-supplement-Suppl__Figure_2.png]
